# Rectal Carriage of Sequence Type 307 *Klebsiella pneumoniae* High‐Risk Clone Harboring Multiple Carbapenemase Genes in Community Hospitals Gauteng, South Africa

**DOI:** 10.1002/mbo3.70152

**Published:** 2025-11-14

**Authors:** Kafilat Taiwo Salvador‐Oke, Johann Pitout, Gisele Peirano, Kathy‐Anne Strydom, Chanel Kingsburgh, Marthie Ehlers, Marleen Kock

**Affiliations:** ^1^ Department of Medical Microbiology, Faculty of Health Sciences University of Pretoria Pretoria South Africa; ^2^ Department of Pathology and Laboratory Medicine University of Calgary Calgary Canada; ^3^ Alberta Precision Laboratories Calgary Canada; ^4^ National Reference Laboratory, Ampath Centurion South Africa; ^5^ Department of Medical Microbiology, Tshwane Academic Division National Health Laboratory Service Pretoria South Africa

**Keywords:** *bla*_OXA‐181_, carbapenem‐resistant *Klebsiella pneumoniae*, high‐risk clones, IncX3 plasmid, rectal colonization, ST307

## Abstract

Asymptomatic rectal carriers are recognized as reservoirs of carbapenem‐resistant *Klebsiella pneumoniae* (CRKp), which can spread epidemic high‐risk clones [e.g., sequence types (ST)‐307] and plasmids [incompatibility group (Inc)‐X3] in hospitals, with possible transmission into the community. This study investigated the epidemiology and characteristics of CRKp high‐risk clones ST307 among rectal carriage isolates from community hospitals. A carbapenemase positivity rate of 24% was observed for all rectal screening performed during hospital admission (February to August 2021) in Gauteng, South Africa; 252 CRKp isolates were characterized. Antimicrobial susceptibility was performed using the VITEK 2 automated system, and polymerase chain reaction assays were used to detect *K. pneumoniae* ST307, carbapenemase genes, and associated mobile genetic elements (MGEs e.g., IncX3, IS3000). Of the 252 isolates, 25% (64/252) were ST307 positive and 75% (188/252) were non‐ST307. Among the 64 ST307, 45% (29/64) harbored *bla*_OXA‐181_ on IncX3 plasmids. Occurrence of *bla*_OXA‐181_ among ST307 (69%; 44/64) when compared to non‐ST307 (48%; 91/188) was statistically significant (*p*‐value = 0.002). Fourteen isolates, including two ST307, harbored double carbapenemase genes. Carbapenemase gene combinations include six *bla*_NDM_ *+bla*_OXA‐48‐like_, four *bla*_NDM_ *+bla*_OXA‐181_, three *bla*_KPC_ *+bla*_OXA‐181_, and one *bla*_OXA‐181_+*bla*_VIM_. One ST307 isolate harbored three carbapenemase genes (*bla*_NDM_ *+bla*_OXA‐48_ *+bla*_OXA‐181_). Level of antimicrobial resistance was significantly (*p*‐value < 0.001) associated with the occurrence of ST307, comprising 73% (47/64) extensively drug resistant. This study highlights the need for rectal screening of XDR clones and plasmids using simple and cost‐effective genomic methodologies suitable for low‐ and middle‐income countries for local risk management and control of infectious diseases in hospitals.

## Graphical abstract

Graphical presentation of rectal carriage of carbapenem‐resistant *Klebsiella pneumoniae*, highlighting ST307 high‐risk clone and IncX3 plasmid‐associated *bla*_OXA‐181._ This study highlights the threat linked to horizontal gene transfer of AMR genes by epidemic plasmids from *Klebsiella pneumoniae* high‐risk clones to other bacterial species.

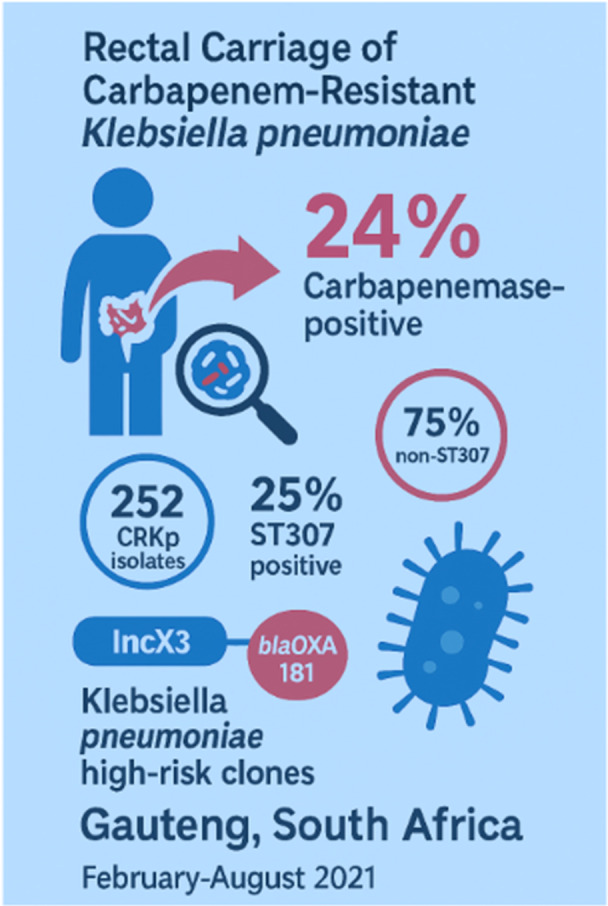

## 1 Introduction

*Klebsiella pneumoniae* high‐risk clones, like sequence type (ST)‐307, have emerged as a public health threat due to their association with the spread of antimicrobial resistance (AMR) (Peirano et al. 2020). This global high‐risk clone possesses increased virulence, survival capabilities, and resistance to numerous antimicrobials, which pose challenges to the management and treatment of associated infections (Peirano et al. 2020). The fitness, persistence, and survival of *K. pneumoniae* ST307 within the hospital environment is due to the presence of specific traits such as capsular, unusual p‐fimbria, plasmid‐mediated glycogen system, and siderophore carried by integrative and conjugative elements (ICEs), a self‐transmissible mobile genetic element (MGE; Villa et al. 2017).

Carbapenemase genes, particularly *K. pneumoniae* carbapenemase (*bla*_KPC_), New Delhi metallo‐β‐lactamase (*bla*_NDM_), oxacillinase (*bla*_OXA‐48‐like_), and Verona integron‐encoded metallo‐β‐lactamase (*bla*_VIM_), are the major cause of resistance to carbapenems and other β‐lactam antimicrobials (Bonomo et al. 2018; Pitout et al. 2015).

The gastrointestinal tract of individuals can be colonized asymptomatically by *K. pneumoniae* high‐risk clones (Stercz et al. 2024). Persistent colonization might lead to difficult‐to‐treat infections due to its association with AMR, particularly resistance to last‐resort antimicrobials such as colistin and carbapenems (Stercz et al. 2024). Rectal colonization with *K. pneumoniae* in asymptomatic carriers is recognized as a reservoir of carbapenem‐resistant *K. pneumoniae* (CRKp) in hospitals and plays an important role in its transmission, possibly into the community (Dao et al. 2014).

Spread of carbapenemase genes is mostly associated with MGEs such as plasmids (Budia‐Silva et al. 2024). Epidemic plasmids like incompatibility (Inc)‐X3 plasmids, which are a globally prevalent plasmid type, enhance the spread of carbapenemase genes [e.g., *bla*_NDM_, *bla*_OXA‐181_] via horizontal gene transfer (HGT) between different bacterial genera, bacterial strains, and other plasmids (Guo et al. 2022). This is due to the plasmid's low fitness cost, high stability, and transmissibility (Pitout et al. 2019). Presence of IncX3 plasmids is associated with reduced susceptibility to clinically applicable antimicrobials, which can lead to treatment failure and infections linked to high morbidity and mortality rates (Chmelnitsky et al. 2013; Juraschek et al. 2021).

The presence of carbapenem‐resistant ST307 *K. pneumoniae* high‐risk clone among inpatients has been documented in South Africa (Lowe et al. 2019; Strydom et al. 2020; Salvador‐Oke et al. 2024a, 2024b). Limited information is available on the rectal colonization of CRKp and the associated high‐risk clone ST307 within the South African hospital and community settings.

The aim of this study was to investigate the prevalence, clonal distribution, antimicrobial resistance mechanisms, and clinical implications of CRKp (particularly ST307 compared with other clones) in rectal carriage isolates of inpatients from community hospitals to understand *K. pneumoniae* epidemiology, risk factors for colonization, and its potential role in subsequent infection and transmission. This study investigated the epidemiology and characteristics of carbapenem‐resistant ST307 and other *K. pneumoniae* clones in rectal carriage of inpatients from community hospitals in South Africa using simple, cost‐effective polymerase chain reaction (PCR) assays. These methods are useful in low‐ and middle‐income countries (LMICs), where genomic surveillance tools are not easily accessible.

## 2 Materials and Methods

### 2.1 Study Design and Bacterial Isolate Collection

This descriptive study investigated the epidemiology and characteristics of CRKp obtained from the rectal screening of inpatients from community hospitals. A 24% carbapenemase positivity rate was reported by the laboratory for all rectal carriage isolates screened during hospital admission or stay between February and August 2021. Isolates were collected across five provinces (Free States, Gauteng, Limpopo, Mpumalanga, and Northwest) of South Africa, with the majority from Gauteng province. The patients were selected as part of a routine infection prevention control (IPC) screening strategy. Isolates were screened for the presence of carbapenemases using the Chrom‐ID CARBA SMART agar (bioMérieux, Marcy l’Étoile, France) according to the manufacturer's instructions.

### 2.2 Microbial Identification and Demographic Data

Microbial identification of cultured isolates was confirmed using the matrix‐assisted laser desorption ionization time‐of‐flight mass spectrometry (MALDI‐TOF MS, Bruker Daltonics, US) at the Ampath National Reference Laboratory (Ampath‐MDRC), Pretoria. Further analyses were performed on the carbapenemase‐positive *K. pneumoniae* isolates at the Medical Microbiology Department, Faculty of Health Sciences, University of Pretoria (UP). Patients' demographic data (age, gender, location, screening date, and date of admission to ward) were collected from the Ampath National Reference Laboratory (Ampath‐MDRC).

### 2.3 Antimicrobial Susceptibility Testing

Rectal carriage isolates were subjected to antimicrobial susceptibility testing (AST GN255) using the VITEK 2 automated system (bioMérieux‐Vitek, Marcy‐l’Étoile, France) according to manufacturer's instructions. *Staphylococcus epidermidis* at 0.50–0.63 McFarland was used as the American Type Culture Collection (ATCC) control strain. The antimicrobial panel consisted of 19 antimicrobials from nine antimicrobial classes. Results were interpreted according to the Clinical and Laboratory Standards Institute breakpoints (CLSI 2020). Colistin resistance isolates from the VITEK 2 results were confirmed using polymyxin NP test (Nordmann et al. 2016) and broth microdilution (EUCAST 2016). Standard definitions were used for multidrug‐resistant, extensively drug‐resistant (XDR), and pandrug‐resistant (PDR) bacteria (Magiorakos et al. 2012). *K. pneumoniae* isolates non‐susceptible to at least one antimicrobial agent in more than three antimicrobial categories were defined as MDR. *K. pneumoniae* isolates non‐susceptible to at least one agent in all but two or fewer antibiotic categories are XDR, while PDR are isolates non‐susceptible to all tested antibiotics.

### 2.4 Molecular Detection of Carbapenemase‐Producing *Klebsiella pneumoniae* ST307 From Rectal Carriage Isolates

Genomic DNA extraction was performed on an overnight culture using the boiling method with modifications (Queipo‐Ortuño et al. 2008). Briefly, a single colony of overnight culture was suspended in 1 milliliter (mL) phosphate‐buffered saline (PBS, pH 7.2: Gibco, Thermo Fisher Scientific, US) and centrifuged [Spectrafuge, Labnet International Incorporation (Inc), US] at 6500 relative centrifugal force (×g) for 5 min (min). The supernatant was discarded, and the pellet was resuspended in 50 µL of PBS. The cell suspension was incubated in a heated block [AccuBlockTM Digital Dry Bath, Labnet, International Incorporation (Inc), US] at 95°C for 15 min. Lysis of cells was carried out by sonication with an ultrasonic bath (Transsonic 460, Elma ultrasonic, Germany) at 100 watts (W) for 15 min. Lysate was centrifuged [Spectrafuge, Labnet International Inc, US] at 13,500 *g* for 5 min, and supernatant was stored at −20°C for further analysis (Salvador‐Oke et al. 2024a, 2024b).

All CRKp isolates were screened for the presence of ST307, epidemic plasmid, IncX3, OXA‐181‐insertion sequence (IS)‐3000, OXA‐48‐IS‐1999, and truncated IS1999 MGEs using PCR assays as previously described (Lowe et al. 2019). Isolates not positive for ST307 were classified as non‐ST307 (Salvador‐Oke et al. 2024a, 2024b). Carbapenemase genes (*bla*_NDM_, *bla*_KPC_, *bla*_OXA‐48‐like_, *bla*_IMP_, and *bla*_VIM_) were detected using PCR assays with specific primers as previously described (Poirel et al. 2004, 2011). Both positive (previously confirmed positive samples for ST307 and other genes) and negative (ultra‐pure water) controls were included in all PCR assays.

### 2.5 Data Analysis

Demographic and clinical data of patients (including AST results, carbapenemase gene identity, screening date, location, gender, and age) screened for CRKp colonization were processed using SPSS statistical software version 28.0.0 (SPSS Incorporation, Chicago, IL, US). Continuous variables were presented as mean ± standard deviation (SD). Age was stratified across five categories (≤ 18, 19–37, 38–55, 56–74, and ≥ 75). Categorical variables were presented as numbers and percentages. Fisher's exact test was used for comparison of categorical data such that the prevalence of *K. pneumoniae* high‐risk clones among rectal carriage isolates was stratified by age, gender, and AMR pattern. Two‐sample proportion test was used to compare the molecular profile of ST307 and non‐ST307 clones. Data were considered statistically significant if *p* < 0.05 was achieved at a 95% confidence interval.

## 3 Results and Discussion

### 3.1 Characteristics of Rectal Carriage Isolates

The emergence and spread of CRKp high‐risk clones and epidemic resistance plasmids associated with AMR in both hospital and community settings is of global public health concern (Peirano et al. 2020). In this study, 24% carbapenemase positive was reported for all patients screened for rectal carriage. We characterized 252 carbapenem‐resistant rectal carriage isolates of inpatients from community hospitals in Gauteng, South Africa, using an accurate, rapid, simple, and cost‐effective PCR assay based on the presence of carbapenem‐resistant ST307 *Klebsiella pneumoniae* clone, the associated carbapenemase genes, and MGEs such as IncX3 plasmid (Lowe et al. 2019; Strydom et al. 2020; Salvador‐Oke et al. 2024a, 2024b). Of these, 64 (25%) were ST307 positive and 188 (75%) were non‐ST307 (Table 1 and Supporting Information S1: Appendix 1). The majority of these isolates were obtained from hospitals within the Gauteng (95%, 239/252) province. One hundred and thirty‐eight (55%) male patients and 114 (45%) female patients were screened for rectal colonization. Study participants' mean age was 54 ± 21 years with an age range between 3 days old and 92 years. The median patient age was 56 years. Sixty‐seven (27%) of the 252 rectal screening were performed within 24 h of admission, including one outpatient. Global reports have shown that the ST307 *K. pneumoniae* high‐risk clone is endemic in South African hospitals (Lowe et al. 2019; Strydom et al. 2020). A previous report showed that all 31 (100%) colonized patients admitted to the intensive care unit of a public hospital in KwaZulu‐Natal province (South Africa) harbored ST307 *K. pneumoniae* (Madni et al. 2021). The risk of acquiring ST307 among patients might be due to underlying diseases, long‐term hospitalization, and previous exposure to antimicrobials. Data obtained from this current study suggested the possible successful dispersion of the ST307 *Klebsiella pneumoniae* clone into communities within the study settings.

**Table 1:** Characteristics of carbapenemase‐producing ST307 *Klebsiella pneumoniae* from rectal carriage isolates.^a^

Characteristics	ST307 (*n* = 64; 25%)	Non‐ST307 (*n* = 188; 75%)	Total (*n* = 252)	*p* value
Patient details				
Mean age	54 yrs	54 yrs	—	0.027*
Female	27 (42%)	87 (46%)	114 (45%)	0.663
Male	37 (58%)	101 (54%)	138 (55%)	—
Not susceptible (intermediate or resistant)				
IPM	64 (100%)	178 (95%)	242 (96%)	—
ETP	64 (100%)	180 (96%)	244 (97%)	—
MEM	51 (80%)	98 (52%)	149 (59%)	—
TZP	64 (100%)	186 (99%)	250 (99%)	—
CXM1	64 (100%)	183 (97%)	247 (98%)	—
CXM2	64 (100%)	183 (97%)	247 (98%)	—
AMP	64 (100%)	188 (100%)	252 (100%)	—
AMC	64 (100%)	187 (99%)	251 (99%)	—
FOX	64 (100%)	182 (97%)	246 (98%)	—
CAZ	64 (100%)	181 (97%)	245 (96%)	—
CTX	64 (100%)	181 (97%)	245 (96%)	—
FEP	64 (100%)	181 (97%)	245 (96%)	—
GEN	45 (70%)	117 (62%)	162 (64%)	—
AMK	22 (34%)	62 (33%)	84 (33%)	—
CIP	64 (100%)	176 (94%)	240 (95%)	—
SXT	49 (77%)	113 (60%)	162 (64%)	—
CST	5 (8%)	3 (2%)	8 (3%)	—
TGC	18 (28%)	57 (30%)	75 (30%)	—
NIT	59 (92%)	122 (65%)	181 (72%)	—
Carbapenemase genes:				
OXA‐181 only	44 (69%)	91 (48%)	135 (54%)	0.002*
OXA‐48‐like only	17 (27%)	76 (40%)	93 (37%)	0.069
NDM only	0 (0%)	4 (2%)	4 (2%)	0.368
KPC only	0 (0%)	2 (1%)	2 (1%)	0.646
VIM only	0 (0%)	3 (2%)	3 (1%)	0.578
NDM + OXA‐181	1 (2%)	3 (2%)	4 (2%)	—
KPC + OXA‐181	1 (2%)	2 (1%)	3 (1%)	—
OXA‐181 + VIM	0 (0%)	1 (0.5%)	1 (0.4%)	—
NDM + OXA‐48‐like	0 (0%)	6 (3%)	6 (2%)	—
NDM + OXA‐48 + OXA‐181	1 (2%)	0 (0%)	1 (0.4%)	—
IncX3 plasmids	32 (50%)	67 (36%)	99 (39%)	0.054

^a^ Penicillin: ampicillin (AMP), amoxicillin/clavulanic acid (AMC), piperacillin/tazobactam (TZP), Cephalosporins: cefuroxime (CXM1), cefuroxime‐axetil (CXM2), cefoxitin (FOX), cefotaxime (CTX), ceftazidime, cefepime (FEP), Carbapenem: ertapenem (ETP), imipenem (IPM), meropenem, Aminoglycosides: amikacin (AMK), gentamicin (GEN), ciprofloxacin (CIP), tigecycline (TGC), nitrofurantoin (NIT), colistin (CST), and trimethoprim‐sulfamethoxazole (SXT).

* Statistically significant *p* value = < 0.05.

**Figure 1:**
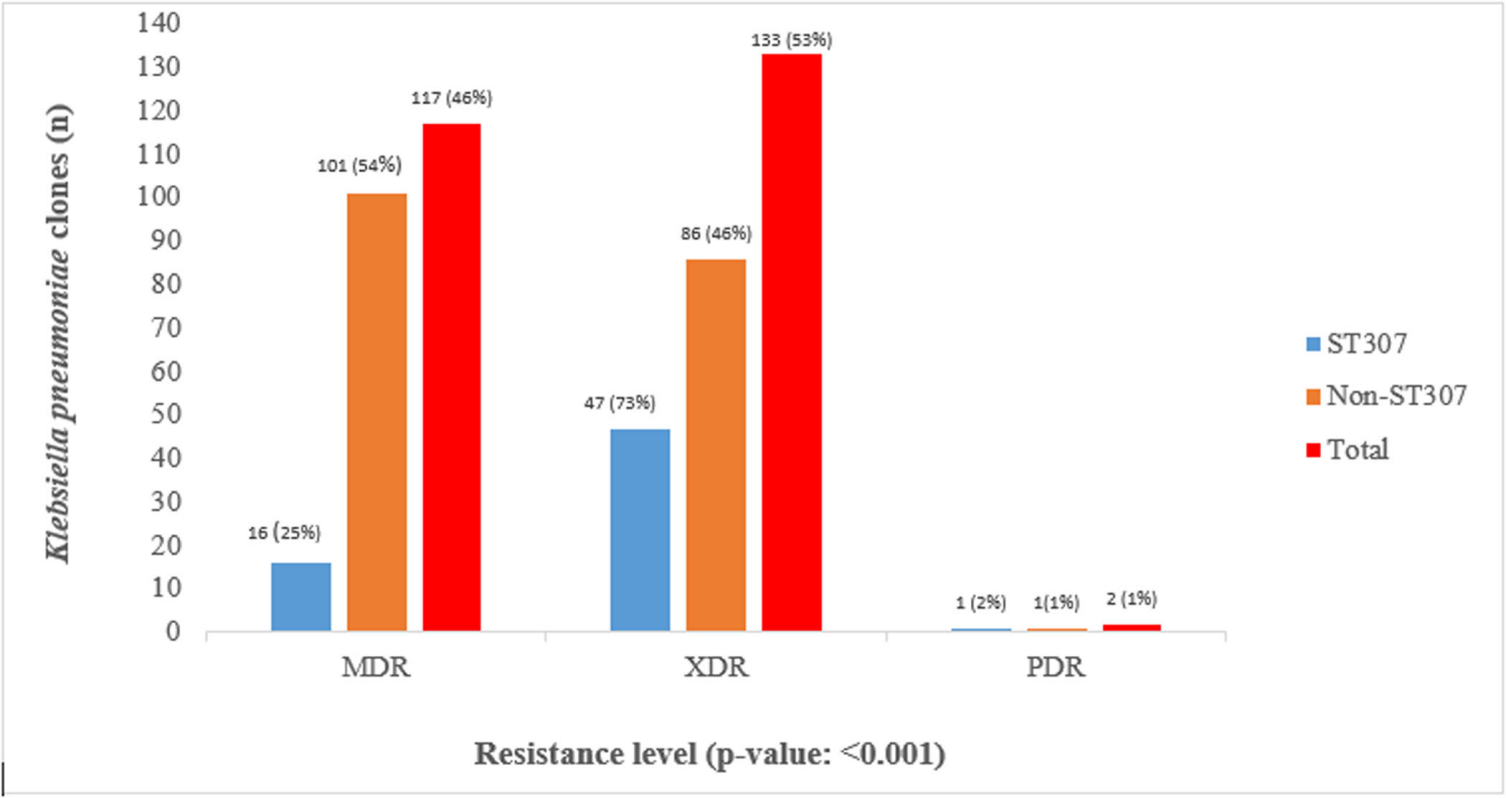
Resistance profile of *Klebsiella pneumoniae* clones among rectal carriage isolates.

The majority of the isolates were XDR, 133 (53%), followed by MDR, 117 (46%), and PDR, 2 (1%) (Figure 1 and Supporting Information S1: Appendix 1). Isolates were mostly resistant to penicillin (99% to 100%), cephalosporins (96% to 98%), carbapenem (59% to 97%), and ciprofloxacin (95%) antimicrobials. Low resistance to colistin was observed in eight (3%) of the 252 isolates (detailed in Supporting Information S1: Appendix 1).

### 3.2 Prevalence and Molecular Characteristics of Carbapenem‐Resistant ST307 *Klebsiella pneumoniae* Among Rectal Carriage Isolates

*Klebsiella pneumoniae* ST307 was identified in patients aged 56 years to 74 years [26 (41%); *p*‐value of 0.027]. Among the 64 ST307 isolates, one (2%) isolate was resistant to all tested antimicrobial classes and 47 (73%) were XDR (Figure 1). The majority of 101 (54%) of the non‐ST307 isolates were MDR, while 86 (46%) were XDR. A statistically significant relationship (*p* < 0.001) was observed between AMR pattern and the occurrence of ST307. Phylogenetic analysis of selected isolates sequenced from this isolate collection revealed that ST307 isolates from this study population were clonally related and possessed similar traits as the South African ST307 from clade VI [e.g., *bla*_OXA‐181_, IncX3, KL102 (*wzi173*), *parC*, and *gyrA*] (Salvador‐Oke et al. 2024a). This provides further evidence that similar ST307 strains are circulating in South Africa. Antimicrobial susceptibility profiles showed that the majority of the carbapenem‐resistant ST307 *K. pneumoniae* isolates exhibited an XDR phenotype. *Klebsiella pneumoniae* ST307 isolates were mostly susceptible to amikacin, tigecycline, and colistin. This is consistent with a previous report in KwaZulu‐Natal province, South Africa, where ICU patients harbored ST307 susceptible to only amikacin and tigecycline (Madni et al. 2021). A report of rectal screening during an outbreak in northeastern Germany demonstrated that ST307 *K. pneumoniae* detected among colonized patients was susceptible to only chloramphenicol, tigecycline, and cefiderocol antimicrobials (Haller et al. 2019).

The *bla*_OXA‐181_ was the most predominant carbapenemase gene detected in 44 (69%) ST307 and 91 (48%) non‐ST307 isolates. Fourteen isolates harbored double carbapenemase genes, including six *bla*_NDM_ *+bla*_OXA‐48_, four *bla*_NDM_ *+bla*_OXA‐181_, three *bla*_KPC_ *+bla*_OXA‐181_, and one *bla*_OXA‐181_+*bla*_VIM_ (Table 1 and Supporting Information S1: Appendix 1). The occurrence of two (*bla*_NDM_ *+bla*_OXA‐181_ and *bla*_KPC_ *+bla*_OXA‐181_) and three (*bla*_NDM_ *+bla*_OXA‐48_+*bla*_OXA‐181_) carbapenemase genes among ST307 (Table 1 and Supporting Information S1: Appendix 1) in this study might pose a significant risk to the efficacy of antimicrobials, as treatment of infections due to *K. pneumoniae* strains that produce carbapenemases is antimicrobial‐specific. The novel β‐lactam/β‐lactamase inhibitors such as imipenem‐relebactam, ceftolozane‐tazobactam, and ceftazidime‐avibactam, which are targeted at the serine‐based carbapenemases (KPC and OXA), will be ineffective in the presence of metallo‐β‐lactamases (NDM, VIM, and IMP; Sakoulas 2019; Van Duin and Bonomo 2016).

The majority of isolates harboring *bla*_OXA‐181_ (*n* = 144) were resistant to carbapenems [ertapenem (*n* = 140), imipenem (*n* = 139), and meropenem (*n* = 85)] but were mostly susceptible to amikacin (*n* = 96), tigecycline (*n* = 101), and colistin (*n* = 141) as seen with the ST307 isolates (Supporting Information S1: Appendix 1).

### 3.3 Association Between ST307 *Klebsiella Pneumoniae*, IncX3, and Carbapenemase Genes Among Rectal Carriage Isolates

A high frequency of the ST307 *K. pneumoniae* isolates (29 (45%) contained a self‐transferable epidemic IncX3‐type plasmid harboring *bla*_OXA‐181_ in this study (Table 2 and Supporting Information S1: Appendix 1). The ST307 isolates, which lacked the IncX3 plasmid, harbored *bla*_OXA‐181_, 18 (28%) and *bla*_OXA‐48‐like_, 14 (22%) genes. The detection of *bla*_OXA‐181_ in isolates without IncX3 suggests mobilization to alternative plasmids or chromosomal integration, highlighting the potential for genomic plasticity and dissemination of this resistance gene (Pitout et al. 2019). Several carbapenemase genes, including *bla*_KPC_, *bla*_NDM_, and *bla*_OXA‐181_, were reportedly linked with the IncX3 plasmid (Lowe et al. 2019; Strydom et al. 2020; Kim et al. 2017; Liu, Guo, et al. 2021). Reports of the spread of *bla*_OXA‐181_ enhanced by ST307 and IncX3 plasmid are not limited to the South African healthcare settings; this was also seen in Angola and China (Lowe et al. 2019; Liu et al. 2015; Kieffer et al. 2016).

**Table 2:** IncX3 plasmid harboring carbapenemase genes among *Klebsiella pneumoniae* strains.

	*bla*_KPC_	*bla*_NDM_	*bla*_OXA‐48‐like_	*bla*_OXA‐181_	*bla*_VIM_
ST307 with IncX3 (*n* = 32)	1 (2%)	2 (3%)	3 (5%)	29 (45%)	0 (0%)
ST307 without IncX3 (*n* = 32)	0 (0%)	0 (0%)	14 (22%)	18 (28%)	0 (0%)
Total ST307 = 64	2 (3%)	2 (3%)	17 (27%)	47 (73%)	0 (0%)
Non‐ST307 with IncX3 (*n* = 67)	2 (1%)	0 (0%)	16 (9%)	51(27%)	1 (1%)
Non‐ST307 without IncX3 (*n* = 121)	2 (1%)	13 (7%)	66 (35%)	46 (24%)	3 (2%)
Total non‐ST307 = 188	2 (2%)	13 (7%)	82 (44%)	97 (52%)	4 (2%)

It was observed that ST307 detection displays a fluctuating but upward trend from February to August 2021. The detection of ST307 was relatively high in February (26%, 36/139), peaking in March (33%, 11/33), dropping during April to May (~17%, 5/30), no isolates collected in June, increasing in July (21%, 3/14), and reaching its peak in August (39%, 5/13). In contrast, IncX3 detection was very high in February (45%, 63/139), dropping sharply during March (21%, 7/33), peaking in April (48%, 11/23), and remaining high through May (40%, 12/30) and July (36%, 5/14) with a sharp decline in August (8%, 1/13). No isolates were collected in June. These trends suggest a possible divergence in the dissemination or persistence of ST307 and IncX3 over time.

The low fitness cost associated with the IncX3 plasmid can provide stability to the host bacteria and aid horizontal transfer of multiple AMR determinants (Guo et al. 2022). Therefore, the high observed rectal carriage rate of IncX3 carrying *bla*_OXA‐181_ linked to ST307 (91%) *K. pneumoniae* is a cause for concern. The presence of IncX3 plasmid in the isolate collection was confirmed using whole genome sequencing (WGS) on selected isolates (*n* = 13) in a previous study (Salvador‐Oke et al. 2024a). The sequenced isolates harbored various MGEs, including IncX3 plasmids as well as AMR and virulence determinants. The ST307 isolate harboring three carbapenemase genes was confirmed to possess IncX3 with the highest number of bacteriophages, which might aid in its acquisition of new AMR traits, and increased virulence via HGT (Salvador‐Oke et al. 2024a). Bacterial strains carrying the IncX3 plasmid can harbor other plasmid types, which can co‐integrate with the IncX3 plasmid and increase the spread of AMR genes (Liu, Wang, et al. 2021). Furthermore, evolutionary changes can occur through the fusion of multiple MDR plasmids (Kieffer et al. 2016). This might result in a stable and effective transmission of AMR genes among bacteria in humans, animals, and the environment, further limiting therapeutic options (Guo et al. 2022).

The dissemination of ST307 associated with IncX3 plasmid carrying *bla*_OXA‐181_ in South Africa has been linked to intrahospital, interhospital, intercity, and interprovince transfer of patients (Lowe et al. 2019). Colonized patients are at increased risk of becoming infected and can inadvertently spread pathogens to others. The spread of *bla*_OXA‐181_ among ST307 isolates has been associated with other plasmid replicons such as ColKP3, IncFIB (K), and IncFII (K) in a previous South African study (Madni et al. 2021). The *bla*_OXA‐181_ among ST307 isolates lacking IncX3 in our study might be associated with one of these other plasmid replicons.

Additionally, 27% (51/188) of the non‐ST307 *Klebsiella pneumoniae* isolates were linked to IncX3 plasmid harboring *bla*_OXA‐181_ (Table 2 and Supporting Information S1: Appendix 1). The *bla*_OXA‐48‐like_ (35%; 66/188) was predominant among non‐ST307, which lacked the IncX3 plasmid. The presence of IncX3 plasmid among the non‐ST307 *K. pneumoniae* isolates in this study highlights the importance of the threat posed by this highly transferable plasmid. A previous study in South Africa reported that after the entry of IncX3 plasmid into a tertiary hospital via ST307 *K. pneumoniae*, it spread to non‐ST307 *K. pneumoniae*, *Escherichia coli*, and *Enterobacter* species (Strydom et al. 2020). Simultaneously, the non‐ST307 *K. pneumoniae* strains accounted for distinct nosocomial outbreaks (Strydom et al. 2020). This highlights the combined effect of *K. pneumoniae* high‐risk clones and epidemic plasmids in the spread of AMR in South Africa. Knowledge of AMR mechanisms is vital to prevent the transmission of AMR genes to other bacterial pathogens of clinical relevance such as *Escherichia coli* (*E. coli*) via HGT (Blázquez et al. 2012; C Reygaert and Department of Biomedical Sciences, Oakland University William Beaumont School of Medicine, Rochester, MI, USA 2018). Considering the present findings, in an endemic setting such as South Africa, implementation of contact precautions for CRKp colonized patients is necessary as a key strategy to prevent person‐to‐person transmission. This will aid optimal CRKp infection prevention and control (IPC) strategies in hospitals and within community settings to reduce the burden of AMR‐associated infections. These precautionary measures have been proposed and shown to reduce the prevalence of CRKp‐associated infections, since the development of CRKp infections has been linked to persistence of CRKp colonization (Haller et al. 2019; Gorrie et al. 2017; Akturk et al. 2016; Martin and Bachman 2018; Qin et al. 2020). Other preventative measures include antimicrobial stewardship, environmental cleaning, and active screening of CRKp colonized patients (Ambretti et al. 2019). Continuous use of rapid molecular methods for the detection of CRKp will reduce the turnaround time for the identification and isolation of colonized patients (Lau et al. 2015). These methods are valuable for low‐ and middle‐income countries, where genomic surveillance methods are not readily affordable.

The major strength of this study is the large sample size (*N* = 252), which suggests that the findings may be used as representative data to guide the IPC of CRKp high‐risk clones among colonized patients in Gauteng, South Africa. This study has a number of limitations including lack of in‐depth genetic analysis, lack of documented history of exposure to the healthcare facilities, which limit study conclusions, and not all patients were screened at admission for CRKp colonization, so carriage cannot be exclusively linked to community settings as some colonization might have occurred before hospitalization, and not within the healthcare facility. Additionally, the exact number of patients screened for rectal carriage and the total *K. pneumoniae* isolates positive for carbapenemase production for the study period was not specified. This was a descriptive study; therefore, it was not possible to follow up patients and examine their progression to CRKp infection. The study population was solely from private hospitals, and the majority of the patients were from the Gauteng province of South Africa, which might have resulted in selection bias. Although the findings from private hospitals provide important insights, they cannot be assumed to be representative of other healthcare sectors in South Africa, especially public hospitals. This is due to differences in the socioeconomic conditions of the patient population, infrastructure, and resource availability, as well as infection control and management practices. A longitudinal study is necessary to investigate *K. pneumoniae* carriage among outpatients visiting general practitioners (GPs) and determine whether patients colonized by *K. pneumoniae* high‐risk clones develop infections due to the CRKp colonizing strains. Genomic characteristics of the colonization isolates are also recommended to provide in‐depth genetic variation of CRKp isolates.

## 4 Conclusion

This study demonstrated that the ST307 represented a quarter of all *K. pneumoniae* detected from rectal carriage of inpatients. However, ST307 was significantly associated with *bla*_OXA‐181_ carriage on epidemic plasmid, IncX3, and a high prevalence of carbapenemase genes as comparable to non‐ST307 clones. The detection of isolates with multiple carbapenemase genes, including a rare ST307 strain harboring three distinct genes, highlights the capacity of this lineage to accumulate diverse resistance determinants. The ST307 clone was generally XDR, underscoring its role as a high‐risk clone in community hospital settings. This study highlights the threat linked to HGT of AMR by epidemic plasmids from *K. pneumoniae* high‐risk clones to other bacterial species. The emergence of ST307 as an XDR clone carrying *bla*_OXA‐181_ on epidemic IncX3 plasmids underscores the potential for HGT to extend beyond hospital settings. Such plasmids act as vehicles for the dissemination of resistance genes into community, veterinary, and environmental reservoirs, highlighting the interconnected threat of AMR within a One Health framework. These findings provide an early warning of cross‐sector dissemination risk and underscore the need to integrate hospital findings into regional One Health AMR surveillance across human, animal, and environmental reservoirs. Continuous genomic surveillance and monitoring of at‐risk patients during hospital admission is critical to ensure timely detection and control of *K. pneumoniae* high‐risk clones and associated plasmids. This strategy would aid local risk management and control of infectious diseases within South African hospitals and the community.

## Author Contributions

**Kafilat Taiwo Salvador‐Oke:** conceptualisation, methodology, formal analysis and investigation, writing – original draft preparation, writing – review and editing. **Johann Pitout:** conceptualisation, methodology, formal analysis and investigation, writing – original draft preparation, writing – review and editing, and supervision. **Gisele Peirano:** writing – review and editing. **Kathy‐Anne Strydom:** writing – review and editing and resources – clinical isolates and patients' data. **Chanel Kingsburgh:** writing – review and editing, and resourses – clinical isolates and patients data. **Marthie Ehlers:** writing – review and editing. **Marleen Kock:** conceptualisation, methodology, formal analysis and investigation, writing – original draft preparation, writing – review and editing, funding acquisition, resources, and supervision.

## Ethics Statement

All procedures were performed in compliance with relevant laws and institutional guidelines and have been approved by the Research Ethics Committee, Faculty of Health Sciences, University of Pretoria, South Africa (Ethics Reference No: 819/2020). The study was performed according to the Declaration of Helsinki. Since clinical bacterial isolates were used for this study, the need for informed consent to participate was waived by the Research Ethics Committee, Faculty of Health Sciences, University of Pretoria, South Africa (Ethics Reference No: 819/2020). All data were anonymized to ensure the privacy and confidentiality of patients' personal information, with each participant assigned a unique identifier.

## Conflicts of Interest

The authors declare no conflicts of interest.

## Supporting information

Supmat.

## Data Availability

All data produced or analyzed during this study are included in this article and its supplementary files.
