# Supplementary material for: Rectal Carriage of Sequence Type 307 Klebsiella pneumoniae High‐Risk Clone Harboring Multiple Carbapenemase Genes in Community Hospitals Gauteng, South Africa
Source: Microbiologyopen. 2025 Nov 14;14(6):e70152. doi: 10.1002/mbo3.70152 (PMC12617265; doi:10.1002/mbo3.70152)
Supplement: Supplementary file 1 — Supmat. [file MBO3-14-e70152-s001.docx]

**Appendix 1: Table A-1 Antimicrobial susceptibility and molecular profiles of rectal carriage isolates^^[[1]](#footnote-1)^^**

|  |  |  | **Antimicrobials** | | | | | | | | | | | | | | | | | | | **Carbapenemase genes** | | | | |  |
| --- | --- | --- | --- | --- | --- | --- | --- | --- | --- | --- | --- | --- | --- | --- | --- | --- | --- | --- | --- | --- | --- | --- | --- | --- | --- | --- | --- |
| **Isolates** | **ST307** | **IncX3** | **AMP** | **AMC** | **TZP** | **CXM1** | **CXM2** | **FOX** | **CTX** | **CAZ** | **FEP** | **ETP** | **IPM** | **MEM** | **AMK** | **GEN** | **CIP** | **TGC** | **NIT** | **CST** | **SXT** | **bla_KPC_** | **bla_NDM_** | **bla_OXA-_**  **_48-like_** | **bla_OXA-_**  **_181_** | **bla_VIM_** | **RES Level** |
| CRKP1 | yes | yes | R | R | R | R | R | R | R | R | R | R | I | R | S | R | R | S | R | S | R | no | no | yes | yes | no | MDR |
| CRKP2 | no | yes | R | R | R | R | R | R | R | R | R | R | R | R | S | S | R | R | R | S | R | no | no | yes | yes | no | XDR |
| CRKP3 | no | no | R | R | R | R | R | R | R | R | R | R | I | S | S | S | R | S | S | S | R | no | no | yes | yes | no | MDR |
| CRKP4 | no | yes | R | R | R | R | R | R | R | R | R | I | I | S | S | S | R | S | S | S | S | no | no | yes | no | no | MDR |
| CRKP5 | no | no | R | R | R | R | R | R | R | R | R | R | R | R | R | R | S | S | I | S | R | no | yes | yes | no | no | MDR |
| CRKP6 | no | no | R | R | R | R | R | R | R | R | R | R | R | S | S | R | R | S | I | S | S | no | no | yes | yes | no | MDR |
| CRKP7 | no | yes | R | R | R | R | R | R | R | R | R | R | I | S | S | S | R | R | I | S | R | no | no | yes | yes | no | XDR |
| CRKP8 | no | no | R | R | R | R | R | R | R | R | R | I | I | S | S | S | R | S | I | S | S | no | no | yes | yes | no | MDR |
| CRKP9 | no | no | R | R | R | R | R | R | R | R | R | R | R | R | S | R | R | I | R | R | S | no | no | yes | no | no | XDR |
| CRKP10 | no | no | R | R | R | R | R | R | R | R | R | R | I | I | S | S | I | S | S | S | R | no | no | yes | no | no | MDR |
| CRKP11 | no | no | R | R | R | R | R | R | R | R | R | R | R | S | I | R | R | I | S | S | R | no | no | yes | yes | no | XDR |
| CRKP12 | no | yes | R | R | R | R | R | R | R | R | R | R | I | I | S | R | R | S | S | S | R | no | no | yes | no | no | MDR |
| CRKP13 | no | no | R | R | R | R | R | R | R | R | R | R | I | S | S | R | R | S | S | S | R | no | no | yes | yes | no | MDR |
| CRKP14 | no | no | R | R | R | R | R | R | R | R | R | R | R | R | R | I | R | R | R | S | R | no | no | yes | no | no | XDR |
| CRKP15 | no | no | R | R | R | R | R | R | R | R | R | R | R | S | S | S | R | R | R | S | R | no | no | yes | no | no | XDR |
| CRKP16 | no | no | R | R | R | R | R | R | R | R | R | R | I | S | I | R | R | S | R | S | R | no | no | yes | yes | no | XDR |
| CRKP17 | no | no | R | R | R | R | R | R | R | R | R | R | R | R | S | S | S | S | S | S | S | no | yes | no | no | no | MDR |
| CRKP18 | no | yes | R | R | R | R | R | R | R | R | R | R | R | R | S | S | R | R | R | S | R | yes | no | yes | yes | no | XDR |
| CRKP19 | no | yes | R | R | R | R | R | R | R | R | R | R | I | S | I | R | R | S | S | S | R | no | no | yes | yes | no | MDR |
| CRKP20 | no | no | R | R | R | R | R | R | R | R | R | R | R | R | R | I | R | R | R | S | R | no | no | yes | no | no | XDR |
| CRKP21 | no | no | R | R | R | R | R | R | R | R | R | R | I | S | S | R | R | S | S | S | S | no | no | yes | yes | no | MDR |
| CRKP22 | no | yes | R | R | R | R | R | R | R | R | R | R | R | I | S | R | R | S | R | S | R | no | no | yes | yes | no | XDR |
| CRKP23 | no | yes | R | R | R | R | R | R | R | R | R | R | I | S | S | R | R | S | S | S | S | no | no | yes | yes | no | MDR |
| CRKP24 | yes | no | R | R | R | R | R | R | R | R | R | R | I | R | I | S | R | I | R | S | S | no | no | yes | yes | no | XDR |
| CRKP25 | no | yes | R | R | R | R | R | R | R | R | R | R | R | S | S | R | R | S | S | S | S | no | no | yes | yes | no | MDR |
| CRKP26 | no | no | R | R | R | R | R | R | R | R | R | R | R | R | I | S | R | I | R | S | R | no | no | yes | yes | no | XDR |
| CRKP27 | no | no | R | R | R | R | R | R | R | R | R | R | I | S | S | R | R | S | R | S | R | no | no | yes | yes | no | XDR |
| CRKP28 | no | no | R | R | R | R | R | R | R | R | R | R | I | S | S | R | R | S | R | S | R | no | no | yes | no | no | XDR |
| CRKP29 | no | no | R | R | R | R | R | R | R | R | R | R | I | R | I | R | R | S | R | S | R | no | no | yes | yes | no | XDR |
| CRKP30 | no | no | R | R | R | R | R | R | R | R | R | R | R | R | I | R | R | S | I | S | R | no | no | yes | no | no | XDR |
| CRKP31 | yes | yes | R | R | R | R | R | R | R | R | R | R | I | R | S | R | R | S | R | S | R | no | no | yes | yes | no | XDR |
| CRKP32 | yes | no | R | R | R | R | R | R | R | R | R | R | R | R | I | R | R | S | R | S | R | no | no | yes | yes | no | XDR |
| CRKP34 | yes | yes | R | R | R | R | R | R | R | R | R | R | R | R | S | S | R | I | R | S | R | no | no | yes | yes | no | XDR |
| CRKP35 | yes | no | R | R | R | R | R | R | R | R | R | R | I | R | S | R | R | R | R | S | R | no | no | yes | yes | no | XDR |
| CRKP36 | no | no | R | R | R | R | R | R | R | R | R | R | R | S | S | R | R | S | I | S | R | no | no | yes | yes | no | XDR |
| CRKP37 | no | no | R | R | R | R | R | R | R | R | R | R | R | R | I | R | R | S | I | S | R | no | no | no | no | yes | XDR |
| CRKP38 | yes | no | R | R | R | R | R | R | R | R | R | R | I | R | S | R | R | S | R | S | R | no | no | yes | yes | no | XDR |
| CRKP40 | yes | no | R | R | R | R | R | R | R | R | R | R | I | R | I | R | R | S | R | S | R | no | no | yes | yes | no | XDR |
| CRKP41 | no | no | R | R | R | R | R | R | R | R | R | I | R | R | S | S | R | S | S | S | R | yes | no | no | no | no | MDR |
| CRKP42 | no | no | R | R | R | R | R | R | R | R | R | R | R | S | S | R | R | R | I | S | R | no | no | yes | yes | no | XDR |
| CRKP43 | yes | no | R | R | R | R | R | R | R | R | R | R | R | R | S | S | R | R | R | R | R | no | no | yes | no | no | XDR |
| CRKP45 | no | no | R | R | R | R | R | R | R | R | R | R | R | S | I | R | R | S | S | S | R | no | no | yes | no | no | MDR |
| CRKP46 | no | no | R | R | R | R | R | R | R | R | R | I | R | R | S | R | S | S | S | S | S | no | no | no | no | yes | MDR |
| CRKP47 | no | no | R | R | R | R | R | R | R | R | R | I | I | S | I | S | R | S | S | S | S | no | no | yes | yes | no | MDR |
| CRKP48 | yes | no | R | R | R | R | R | R | R | R | R | R | R | R | I | S | R | S | R | S | R | no | no | yes | no | no | XDR |
| CRKP49 | no | no | R | R | R | R | R | R | R | R | R | R | I | R | S | R | R | S | R | S | R | no | no | yes | yes | no | XDR |
| CRKP50 | yes | no | R | R | R | R | R | R | R | R | R | R | I | S | S | R | R | S | I | S | R | no | no | yes | yes | no | XDR |
| CRKP51 | no | yes | R | R | R | R | R | R | R | R | R | R | I | S | S | R | R | S | S | S | R | no | no | yes | yes | no | MDR |
| CRKP53 | no | no | R | R | R | R | R | R | R | R | R | R | I | S | S | R | R | S | R | S | R | no | no | yes | yes | no | XDR |
| CRKP54 | no | no | R | R | R | S | S | R | S | S | S | S | S | S | S | S | R | S | S | S | S | no | no | yes | yes | no | MDR |
| CRKP55 | no | no | R | R | R | R | R | R | R | R | R | R | R | R | R | R | R | R | R | S | R | no | yes | yes | no | no | XDR |
| CRKP56 | no | no | R | S | S | R | R | R | S | S | S | S | S | S | S | S | S | S | I | S | S | no | no | yes | no | no | MDR |
| CRKP57 | no | no | R | R | R | R | R | R | R | R | R | R | I | I | I | R | R | S | S | S | R | no | no | yes | no | no | MDR |
| CRKP58 | yes | no | R | R | R | R | R | R | R | R | R | R | R | R | I | R | R | S | R | S | R | no | no | yes | yes | no | XDR |
| CRKP59 | no | yes | R | R | R | R | R | R | R | R | R | R | I | I | I | R | R | S | S | R | S | no | no | yes | yes | no | MDR |
| CRKP60 | no | no | R | R | R | R | R | R | R | R | R | R | I | I | I | R | R | S | S | S | R | no | no | yes | yes | no | MDR |
| CRKP61 | no | no | R | R | R | R | R | R | R | R | R | R | I | S | S | S | S | S | I | S | S | no | no | yes | no | no | MDR |
| CRKP62 | yes | yes | R | R | R | R | R | R | R | R | R | R | I | R | S | R | R | R | R | S | R | no | no | yes | yes | no | XDR |
| CRKP63 | yes | yes | R | R | R | R | R | R | R | R | R | R | I | R | S | R | R | R | R | S | R | no | no | yes | yes | no | XDR |
| CRKP64 | no | yes | R | R | R | R | R | R | R | R | R | I | R | R | S | S | R | S | S | S | S | no | no | yes | yes | yes | MDR |
| CRKP65 | no | yes | R | R | R | R | R | R | R | R | R | R | I | S | S | S | R | S | S | S | R | no | no | yes | yes | no | MDR |
| CRKP66 | no | yes | R | R | R | R | R | S | R | R | R | R | S | S | S | S | R | S | I | S | S | no | no | yes | yes | no | MDR |
| CRKP67 | no | yes | R | R | R | R | R | R | R | R | R | R | I | S | S | S | R | S | S | S | S | no | no | yes | yes | no | MDR |
| CRKP68 | no | yes | R | R | R | R | R | R | R | R | R | R | R | R | S | S | R | S | R | S | R | no | no | yes | yes | no | MDR |
| CRKP69 | no | yes | R | R | R | R | R | R | R | R | R | R | I | S | S | R | R | S | S | S | S | no | no | yes | no | no | MDR |
| CRKP70 | no | yes | R | R | R | R | R | R | R | R | R | R | R | S | S | R | R | S | S | S | S | no | no | yes | yes | no | MDR |
| CRKP71 | no | yes | R | R | R | R | R | R | R | R | R | R | R | S | S | S | R | S | R | S | S | no | no | yes | yes | no | MDR |
| CRKP72 | no | yes | R | R | R | R | R | R | R | R | R | R | R | S | S | R | R | S | S | S | R | no | no | yes | yes | no | MDR |
| CRKP73 | yes | yes | R | R | R | R | R | R | R | R | R | R | R | R | R | R | R | S | R | S | R | no | yes | yes | yes | no | XDR |
| CRKP74 | yes | yes | R | R | R | R | R | R | R | R | R | R | I | R | S | R | R | S | R | S | R | no | no | yes | yes | no | XDR |
| CRKP75 | yes | yes | R | R | R | R | R | R | R | R | R | R | I | R | S | R | R | S | R | S | R | no | no | yes | no | no | XDR |
| CRKP76 | yes | yes | R | R | R | R | R | R | R | R | R | R | I | S | S | R | R | S | S | S | S | no | no | yes | yes | no | MDR |
| CRKP77 | yes | yes | R | R | R | R | R | R | R | R | R | R | R | S | S | S | R | S | S | S | R | no | no | yes | yes | no | MDR |
| CRKP79 | yes | no | R | R | R | R | R | R | R | R | R | R | I | I | S | R | R | S | I | S | S | no | no | yes | no | no | MDR |
| CRKP80 | yes | no | R | R | R | R | R | R | R | R | R | R | R | R | S | R | R | S | R | R | S | no | no | yes | no | no | XDR |
| CRKP81 | yes | yes | R | R | R | R | R | R | R | R | R | R | I | R | S | R | R | S | R | S | R | no | no | yes | yes | no | XDR |
| CRKP83 | yes | no | R | R | R | R | R | R | R | R | R | R | I | R | I | R | R | S | R | S | R | no | no | yes | yes | no | XDR |
| CRKP84 | yes | yes | R | R | R | R | R | R | R | R | R | R | R | R | S | R | R | S | R | R | S | no | no | yes | yes | no | XDR |
| CRKP85 | yes | yes | R | R | R | R | R | R | R | R | R | R | R | R | R | R | R | S | R | S | R | yes | no | yes | yes | no | XDR |
| CRKP86 | yes | no | R | R | R | R | R | R | R | R | R | R | I | R | I | R | R | S | R | S | R | no | no | yes | yes | no | XDR |
| CRKP88 | no | no | R | R | R | R | R | R | R | R | R | R | R | R | S | R | R | S | R | S | R | no | no | yes | no | no | XDR |
| CRKP89 | yes | yes | R | R | R | R | R | R | R | R | R | R | I | I | S | S | R | S | I | S | S | no | no | yes | yes | no | MDR |
| CRKP90 | yes | yes | R | R | R | R | R | R | R | R | R | R | I | R | S | R | R | S | R | S | S | no | no | yes | yes | no | MDR |
| CRKP91 | no | no | R | R | R | R | R | R | R | R | R | R | R | R | S | S | R | S | S | S | S | no | no | yes | yes | no | MDR |
| CRKP92 | no | no | R | R | R | R | R | R | R | R | R | R | I | I | I | R | R | R | S | S | R | no | no | yes | yes | no | XDR |
| CRKP93 | no | no | R | R | R | R | R | R | R | R | R | R | R | R | I | R | R | S | I | S | R | no | no | yes | no | no | XDR |
| CRKP94 | no | yes | R | R | R | R | R | R | R | R | R | R | I | S | S | R | R | S | S | S | S | no | no | yes | yes | no | MDR |
| CRKP95 | no | no | R | R | R | R | R | R | R | R | R | R | R | R | I | R | R | S | R | S | R | no | no | yes | yes | no | XDR |
| CRKP96 | no | yes | R | R | R | R | R | R | R | R | R | R | I | I | I | R | R | S | S | S | R | no | no | yes | yes | no | MDR |
| CRKP97 | no | no | R | R | R | R | R | R | R | R | R | R | R | S | S | S | S | S | S | S | S | no | no | yes | no | no | MDR |
| CRKP98 | no | yes | R | R | R | R | R | R | R | R | R | R | I | S | S | R | R | S | S | S | S | no | no | yes | yes | no | MDR |
| CRKP99 | no | yes | R | R | R | R | R | R | R | R | R | R | R | S | I | R | R | R | R | S | R | no | no | yes | yes | no | XDR |
| CRKP101 | no | yes | R | R | R | R | R | R | R | R | R | R | R | R | I | R | R | R | R | S | R | no | no | yes | yes | no | XDR |
| CRKP102 | no | yes | R | R | R | R | R | R | R | R | R | R | R | S | S | S | R | R | I | S | S | no | no | yes | yes | no | MDR |
| CRKP103 | yes | yes | R | R | R | R | R | R | R | R | R | R | I | R | S | R | R | R | R | S | R | no | no | yes | yes | no | XDR |
| CRKP104 | yes | yes | R | R | R | R | R | R | R | R | R | R | I | R | S | R | R | S | R | S | R | no | no | yes | yes | no | XDR |
| CRKP105 | no | no | R | R | R | R | R | R | R | R | R | R | R | R | R | R | R | R | R | R | R | no | no | yes | no | no | PDR |
| CRKP106 | no | no | R | R | R | R | R | R | R | R | R | R | R | R | R | I | R | R | R | S | R | no | no | yes | no | no | XDR |
| CRKP107 | no | yes | R | R | R | R | R | R | R | R | R | R | R | R | I | R | R | S | R | S | R | no | no | yes | yes | no | XDR |
| CRKP108 | no | yes | R | R | R | R | R | R | R | R | R | R | R | R | I | S | R | S | R | S | R | no | no | yes | yes | no | XDR |
| CRKP109 | no | yes | R | R | R | R | R | R | R | R | R | R | R | R | S | S | R | S | R | S | R | no | no | yes | yes | no | MDR |
| CRKP110 | no | no | R | R | R | R | R | R | R | R | R | R | R | S | S | S | R | S | S | S | S | no | no | yes | no | no | MDR |
| CRKP111 | no | yes | R | R | R | R | R | R | R | R | R | R | I | S | S | S | R | S | S | S | S | no | no | yes | yes | no | MDR |
| CRKP112 | no | no | R | R | R | R | R | R | R | R | R | R | R | R | I | S | R | R | R | S | R | no | no | yes | no | no | XDR |
| CRKP113 | yes | yes | R | R | R | R | R | R | R | R | R | R | I | R | I | R | R | S | R | S | R | no | no | yes | yes | no | XDR |
| CRKP114 | yes | yes | R | R | R | R | R | R | R | R | R | R | R | R | R | R | R | S | R | S | R | no | no | yes | yes | no | XDR |
| CRKP115 | no | no | R | R | R | R | R | R | R | R | R | R | R | R | I | S | R | R | R | S | R | no | no | yes | no | no | XDR |
| CRKP116 | no | yes | R | R | R | R | R | R | R | R | R | R | I | S | S | R | R | S | S | S | R | no | no | yes | yes | no | MDR |
| CRKP117 | no | yes | R | R | R | R | R | R | S | S | S | S | S | S | S | S | R | S | S | S | S | no | no | yes | yes | no | MDR |
| CRKP118 | no | no | R | R | R | R | R | R | R | R | R | R | R | R | R | I | R | R | R | S | R | no | yes | yes | no | no | XDR |
| CRKP119 | no | yes | R | R | R | R | R | R | R | R | R | R | I | I | S | S | R | S | S | S | S | no | no | yes | yes | no | MDR |
| CRKP120 | no | no | R | R | R | R | R | R | R | R | R | R | R | R | I | S | R | R | R | S | R | no | yes | yes | yes | no | XDR |
| CRKP121 | no | yes | R | R | R | R | R | R | R | R | R | R | I | R | S | S | R | S | I | S | R | no | no | yes | yes | no | MDR |
| CRKP122 | no | no | R | R | R | R | R | R | R | R | R | R | I | S | S | S | R | S | R | S | R | no | no | yes | no | no | MDR |
| CRKP123 | no | no | R | R | R | R | R | R | R | R | R | R | I | S | S | R | R | S | S | S | S | no | no | yes | yes | no | MDR |
| CRKP124 | no | yes | R | R | R | R | R | R | R | R | R | R | R | S | S | S | R | S | S | S | R | no | no | yes | yes | no | MDR |
| CRKP125 | no | no | R | R | R | R | R | R | R | R | R | R | I | S | S | R | R | S | S | S | S | no | no | yes | no | no | MDR |
| CRKP126 | no | no | R | R | R | R | R | R | R | R | R | R | R | R | I | R | R | S | R | S | R | no | yes | yes | yes | no | XDR |
| CRKP127 | no | no | R | R | R | R | R | R | R | R | R | R | I | S | S | R | R | S | S | S | S | no | no | yes | no | no | MDR |
| CRKP128 | no | yes | R | R | I | S | S | R | S | S | S | S | S | S | S | S | R | S | S | S | S | no | no | yes | yes | no | MDR |
| CRKP129 | no | yes | R | R | R | S | S | S | S | S | S | S | S | S | S | S | R | S | I | S | S | no | no | yes | yes | no | MDR |
| CRKP130 | no | yes | R | R | R | R | R | R | R | R | R | R | I | S | I | R | R | S | S | S | R | no | no | yes | yes | no | MDR |
| CRKP131 | yes | no | R | R | R | R | R | R | R | R | R | R | R | R | I | S | R | S | R | S | R | no | no | yes | yes | no | XDR |
| CRKP132 | no | yes | R | R | R | R | R | R | R | R | R | R | R | R | I | R | R | R | R | S | R | no | no | yes | yes | no | XDR |
| CRKP134 | no | no | R | R | R | R | R | R | R | R | R | R | R | R | S | S | R | R | R | S | R | no | yes | no | no | no | XDR |
| CRKP135 | yes | yes | R | R | R | R | R | R | R | R | R | R | R | R | I | S | R | I | R | R | R | no | no | yes | no | no | PDR |
| CRKP136 | yes | yes | R | R | R | R | R | R | R | R | R | R | R | R | I | R | R | R | R | S | R | no | no | yes | yes | no | XDR |
| CRKP137 | no | no | R | R | R | R | R | R | R | R | R | R | R | S | I | S | S | R | I | S | S | no | no | yes | no | no | MDR |
| CRKP139 | yes | no | R | R | R | R | R | R | R | R | R | R | I | R | S | R | R | S | R | S | R | no | no | yes | yes | no | XDR |
| CRKP140 | no | yes | R | R | R | R | R | R | R | R | R | R | R | R | S | R | R | R | R | S | R | no | no | yes | yes | no | XDR |
| CRKP141 | no | no | R | R | R | R | R | R | R | R | R | R | I | S | S | R | R | S | S | S | S | no | no | yes | no | no | MDR |
| CRKP142 | no | no | R | R | R | R | R | R | R | R | R | R | I | S | I | S | R | S | S | S | R | no | no | yes | no | no | MDR |
| CRKP143 | no | no | R | R | R | R | R | R | R | R | R | R | I | S | S | R | R | S | S | S | S | no | no | yes | no | no | MDR |
| CRKP145 | no | yes | R | R | R | R | R | R | R | R | R | R | R | R | S | R | R | S | R | S | R | no | no | yes | yes | no | XDR |
| CRKP146 | no | no | R | R | R | R | R | R | R | R | R | R | R | R | I | R | R | R | R | S | R | no | no | yes | no | no | XDR |
| CRKP147 | no | yes | R | R | R | R | R | R | R | R | R | R | R | S | S | R | R | S | I | S | R | no | no | yes | no | no | XDR |
| CRKP148 | no | no | R | R | R | R | R | R | R | R | R | R | I | S | S | S | S | S | I | S | S | no | no | yes | no | no | MDR |
| CRKP149 | no | no | R | R | R | R | R | R | R | R | R | R | R | R | S | S | R | R | R | S | R | no | yes | no | no | no | XDR |
| CRKP150 | no | no | R | R | R | R | R | R | R | R | R | R | I | R | S | R | R | S | R | S | R | no | yes | yes | yes | no | XDR |
| CRKP152 | no | no | R | R | R | R | R | R | R | R | R | R | R | R | S | S | R | R | R | S | R | no | no | yes | yes | no | XDR |
| CRKP153 | no | no | R | R | R | R | R | R | R | R | R | R | R | R | S | S | R | I | R | S | R | no | no | yes | yes | no | XDR |
| CRKP154 | no | no | R | R | R | R | R | R | R | R | R | R | R | R | S | S | R | S | S | S | S | no | no | yes | no | no | MDR |
| CRKP155 | no | no | R | R | R | R | R | R | R | R | R | R | R | S | S | R | R | R | I | S | S | no | no | yes | no | no | XDR |
| CRKP156 | no | no | R | R | R | R | R | R | R | R | R | R | R | R | S | R | R | S | R | S | R | no | no | yes | yes | no | XDR |
| CRKP158 | no | no | R | R | R | R | R | R | R | R | R | R | R | S | S | R | R | S | S | S | S | no | no | yes | no | no | MDR |
| CRKP159 | no | no | R | R | R | R | R | R | R | R | R | R | R | R | S | R | R | R | R | S | R | no | no | yes | yes | no | XDR |
| CRKP160 | no | no | R | R | R | R | R | R | R | R | R | R | R | R | S | S | R | S | R | S | S | no | no | yes | no | no | MDR |
| CRKP162 | no | no | R | R | R | R | R | R | R | R | R | R | R | R | R | S | R | R | R | S | R | no | yes | yes | no | no | XDR |
| CRKP163 | yes | no | R | R | R | R | R | R | R | R | R | R | R | S | S | S | R | R | R | S | R | no | no | yes | yes | no | XDR |
| CRKP164 | no | no | R | R | R | R | R | R | R | R | R | R | I | R | S | R | R | S | I | S | S | no | no | yes | no | no | MDR |
| CRKP165 | no | yes | R | R | R | R | R | R | R | R | R | R | I | R | S | S | R | S | R | S | S | no | no | yes | no | no | MDR |
| CRKP166 | yes | no | R | R | R | R | R | R | R | R | R | R | I | S | S | R | R | S | S | S | R | no | no | yes | yes | no | MDR |
| CRKP167 | no | no | R | R | R | R | R | R | R | R | R | R | I | R | I | R | R | R | R | S | S | no | no | yes | yes | no | XDR |
| CRKP168 | yes | no | R | R | R | R | R | R | R | R | R | R | R | R | S | S | R | R | R | S | S | no | no | yes | no | no | MDR |
| CRKP170 | yes | yes | R | R | R | R | R | R | R | R | R | R | I | S | S | R | R | S | S | S | S | no | no | yes | yes | no | MDR |
| CRKP171 | yes | no | R | R | R | R | R | R | R | R | R | R | R | S | S | S | R | S | I | S | S | no | no | yes | yes | no | MDR |
| CRKP172 | yes | no | R | R | R | R | R | R | R | R | R | R | R | R | I | R | R | S | R | S | R | no | no | yes | yes | no | MDR |
| CRKP174 | no | no | R | R | R | R | R | R | R | R | R | R | I | S | S | R | R | S | S | S | R | no | no | yes | no | no | MDR |
| CRKP175 | no | yes | R | R | R | R | R | R | R | R | R | R | R | R | S | S | R | S | R | S | R | yes | no | yes | yes | no | MDR |
| CRKP176 | no | no | R | R | R | R | R | R | R | R | R | R | I | S | S | R | R | R | I | S | R | no | no | yes | no | no | XDR |
| CRKP177 | yes | yes | R | R | R | R | R | R | R | R | R | R | R | R | S | S | R | R | R | I | R | no | no | yes | yes | no | XDR |
| CRKP178 | no | yes | R | R | R | R | R | R | R | R | R | R | R | S | S | S | R | R | I | S | S | no | no | yes | yes | no | MDR |
| CRKP179 | no | no | R | R | R | R | R | R | R | R | R | R | I | S | S | R | R | S | I | S | R | no | no | yes | no | no | XDR |
| CRKP180 | no | no | R | R | R | R | R | R | R | R | R | R | I | R | I | R | R | R | R | S | R | no | no | yes | yes | no | XDR |
| CRKP181 | yes | no | R | R | R | R | R | R | R | R | R | R | I | R | S | S | R | S | R | S | R | no | no | yes | no | no | MDR |
| CRKP182 | no | no | R | R | R | R | R | R | R | R | R | R | I | R | S | R | R | S | R | S | R | no | no | yes | yes | no | XDR |
| CRKP183 | yes | yes | R | R | R | R | R | R | R | R | R | R | R | S | S | R | R | S | I | S | R | no | no | yes | yes | no | XDR |
| CRKP184 | no | no | R | R | R | R | R | R | R | R | R | R | R | R | R | R | R | S | I | S | R | no | yes | no | no | no | XDR |
| CRKP185 | yes | no | R | R | R | R | R | R | R | R | R | R | I | R | S | R | R | S | R | S | S | no | no | yes | yes | no | MDR |
| CRKP186 | no | no | R | R | R | R | R | R | R | R | R | R | I | R | S | R | R | R | R | S | R | no | no | yes | yes | no | XDR |
| CRKP187 | yes | yes | R | R | R | R | R | R | R | R | R | R | I | R | I | R | R | R | R | S | S | no | no | yes | yes | no | XDR |
| CRKP188 | no | no | R | R | R | R | R | R | R | R | R | R | R | R | I | R | R | S | I | S | S | no | no | yes | no | no | MDR |
| CRKP189 | no | yes | R | R | R | R | R | R | R | R | R | R | I | S | S | S | R | S | S | S | R | no | no | yes | yes | no | MDR |
| CRKP190 | no | no | R | R | R | R | R | R | R | R | R | R | R | R | R | I | R | R | R | S | R | no | yes | yes | no | no | XDR |
| CRKP191 | no | no | R | R | R | R | R | R | R | R | R | R | R | R | S | S | R | S | R | S | R | no | no | yes | yes | no | MDR |
| CRKP192 | no | no | R | R | R | R | R | R | R | R | R | R | R | S | S | S | R | S | R | S | S | yes | no | no | no | no | MDR |
| CRKP193 | no | yes | R | R | R | R | R | R | R | R | R | R | R | S | S | R | R | S | S | S | S | no | no | yes | no | no | MDR |
| CRKP194 | yes | yes | R | R | R | R | R | R | R | R | R | R | I | R | S | R | R | S | R | S | R | no | no | yes | no | no | XDR |
| CRKP195 | yes | no | R | R | R | R | R | R | R | R | R | R | I | R | S | R | R | S | R | S | R | no | no | yes | no | no | XDR |
| CRKP196 | yes | no | R | R | R | R | R | R | R | R | R | R | R | R | I | R | R | S | R | S | R | no | no | yes | no | no | XDR |
| CRKP197 | no | yes | R | R | R | R | R | R | R | R | R | R | R | S | S | R | R | R | I | S | S | no | no | yes | no | no | XDR |
| CRKP198 | no | no | R | R | R | R | R | R | R | R | R | R | R | S | S | R | R | S | S | S | S | no | no | yes | no | no | MDR |
| CRKP199 | no | no | R | R | R | R | R | R | R | R | R | R | R | R | R | R | R | R | R | S | S | no | no | yes | no | no | XDR |
| CRKP201 | no | yes | R | R | R | R | R | R | R | R | R | R | I | R | I | R | R | S | R | S | R | no | no | yes | no | no | MDR |
| CRKP202 | no | yes | R | R | R | R | R | R | R | R | R | R | R | R | I | R | R | S | I | S | S | no | no | yes | yes | no | XDR |
| CRKP203 | no | no | R | R | R | R | R | R | R | R | R | R | R | R | S | R | R | R | R | S | R | no | no | yes | no | no | XDR |
| CRKP204 | no | yes | R | R | R | R | R | R | R | R | R | R | R | R | I | S | R | I | R | S | R | no | no | yes | yes | no | XDR |
| CRKP205 | no | yes | R | R | R | S | S | S | S | S | S | R | S | S | S | S | R | S | S | S | S | no | no | yes | no | no | MDR |
| CRKP206 | no | no | R | R | R | R | R | R | R | R | R | R | I | S | S | R | R | S | S | S | S | no | no | yes | no | no | MDR |
| CRKP207 | no | no | R | R | R | R | R | R | R | R | R | R | R | S | S | S | R | S | S | S | R | no | no | yes | yes | no | MDR |
| CRKP208 | yes | no | R | R | R | R | R | R | R | R | R | R | R | S | S | R | R | S | S | S | S | no | no | yes | yes | no | XDR |
| CRKP209 | no | yes | R | R | R | R | R | R | R | R | R | R | I | S | S | R | R | S | I | S | R | no | no | yes | yes | no | MDR |
| CRKP210 | no | no | R | R | R | R | R | R | R | R | R | R | R | S | S | R | R | S | S | S | S | no | no | yes | yes | no | MDR |
| CRKP211 | no | yes | R | R | R | R | R | R | R | R | R | R | I | S | S | R | R | S | S | S | S | no | no | yes | no | no | MDR |
| CRKP212 | no | yes | R | R | R | R | R | R | R | R | R | R | I | R | I | R | R | S | R | S | S | no | no | yes | yes | no | MDR |
| CRKP213 | no | yes | R | R | R | R | R | R | R | R | R | R | R | S | S | R | R | S | S | S | S | no | no | yes | no | no | MDR |
| CRKP214 | no | no | R | R | R | R | R | R | R | R | R | R | I | S | S | S | S | S | S | S | S | no | no | yes | no | no | XDR |
| CRKP215 | yes | no | R | R | R | R | R | R | R | R | R | R | R | R | I | S | R | R | R | S | R | no | no | yes | yes | no | XDR |
| CRKP216 | no | no | R | R | R | R | R | R | R | R | R | R | R | R | S | R | R | S | R | S | R | no | no | yes | no | no | MDR |
| CRKP217 | no | yes | R | R | R | R | R | R | R | R | R | R | I | S | S | S | R | S | I | S | S | no | no | yes | no | no | MDR |
| CRKP218 | no | no | R | R | R | R | R | R | R | R | R | R | R | S | S | S | R | S | I | S | S | no | no | yes | yes | no | XDR |
| CRKP219 | no | yes | R | R | R | R | R | R | R | R | R | I | I | S | S | R | R | I | R | S | R | no | no | yes | no | no | MDR |
| CRKP220 | no | no | R | R | R | R | R | R | R | R | R | R | I | I | S | R | R | S | I | S | S | no | no | yes | no | no | XDR |
| CRKP221 | no | yes | R | R | R | R | R | R | R | R | R | R | I | R | S | R | R | R | R | S | R | no | no | yes | yes | no | XDR |
| CRKP222 | no | yes | R | R | R | R | R | R | R | R | R | R | I | S | S | R | R | S | I | S | R | no | no | yes | no | no | XDR |
| CRKP223 | no | no | R | R | R | R | R | R | R | R | R | R | R | R | I | R | R | S | R | S | R | no | no | yes | no | no | XDR |
| CRKP224 | no | yes | R | R | R | R | R | R | R | R | R | R | I | S | S | S | R | S | I | S | S | no | no | yes | yes | no | MDR |
| CRKP225 | no | no | R | R | R | R | R | R | R | R | R | R | I | S | S | S | S | S | S | S | S | no | no | yes | no | no | MDR |
| CRKP226 | no | yes | R | R | R | R | R | R | R | R | R | R | I | S | S | R | R | R | R | S | R | no | no | yes | yes | no | XDR |
| CRKP227 | no | no | R | R | R | R | R | R | R | R | R | R | I | S | S | R | R | S | S | S | S | no | no | yes | yes | no | MDR |
| CRKP228 | no | no | R | R | R | R | R | R | R | R | R | R | R | R | R | I | R | R | R | S | R | no | yes | yes | no | no | XDR |
| CRKP229 | no | yes | R | R | R | R | R | R | R | R | R | R | R | S | S | R | R | S | I | S | R | no | no | yes | yes | no | XDR |
| CRKP230 | yes | yes | R | R | R | R | R | R | R | R | R | R | I | R | I | R | R | I | R | S | R | no | no | yes | yes | no | XDR |
| CRKP231 | no | no | R | R | R | R | R | R | R | R | R | R | R | S | S | R | R | S | R | S | R | no | no | yes | yes | no | XDR |
| CRKP232 | no | no | R | R | R | R | R | R | R | R | R | R | I | I | I | R | R | S | I | S | S | no | no | yes | yes | no | MDR |
| CRKP233 | no | no | R | R | R | S | S | S | S | S | S | S | S | S | S | S | R | S | S | S | S | no | no | yes | no | no | MDR |
| CRKP234 | yes | yes | R | R | R | R | R | R | R | R | R | R | I | R | S | S | R | S | R | S | S | no | no | yes | yes | no | MDR |
| CRKP235 | no | no | R | R | R | R | R | R | R | R | R | R | R | R | R | R | R | I | R | S | S | no | no | yes | yes | no | XDR |
| CRKP236 | yes | no | R | R | R | R | R | R | R | R | R | R | I | R | S | R | R | S | R | S | R | no | no | yes | no | no | XDR |
| CRKP237 | no | no | R | R | R | R | R | R | R | R | R | R | I | R | S | S | R | S | I | S | S | no | no | yes | no | no | MDR |
| CRKP238 | no | no | R | R | R | R | R | R | R | R | R | R | R | S | S | S | R | S | S | S | R | no | no | yes | no | no | MDR |
| CRKP239 | no | yes | R | R | R | R | R | R | R | R | R | R | I | R | I | R | R | S | R | S | R | no | no | yes | yes | no | XDR |
| CRKP240 | no | no | R | R | R | R | R | R | R | R | R | R | I | R | S | S | R | S | R | S | R | no | no | yes | yes | no | MDR |
| CRKP241 | no | no | R | R | R | R | R | R | R | R | R | R | R | S | S | S | R | S | S | S | S | no | no | yes | no | no | MDR |
| CRKP242 | yes | yes | R | R | R | R | R | R | R | R | R | R | R | R | R | R | R | R | R | S | R | no | no | yes | yes | no | XDR |
| CRKP243 | no | yes | R | R | R | R | R | S | R | R | R | S | S | S | R | R | R | S | R | S | R | no | no | yes | no | no | MDR |
| CRKP244 | no | no | R | R | R | R | R | R | R | R | R | I | R | R | R | R | R | I | R | S | R | no | no | yes | yes | no | XDR |
| CRKP245 | no | no | R | R | R | R | R | R | R | R | R | R | R | S | I | R | R | I | I | S | R | no | no | yes | yes | no | XDR |
| CRKP246 | yes | yes | R | R | R | R | R | R | R | R | R | R | I | R | I | S | R | S | R | S | R | no | no | yes | yes | no | XDR |
| CRKP247 | no | yes | R | R | R | R | R | R | R | R | R | R | I | S | S | R | R | S | I | S | S | no | no | yes | yes | no | MDR |
| CRKP248 | no | no | R | R | R | R | R | R | R | R | R | R | R | R | S | R | R | R | R | S | R | no | no | yes | yes | no | XDR |
| CRKP249 | no | no | R | R | R | R | R | R | R | R | R | R | R | R | R | R | S | S | S | S | R | no | no | no | no | yes | MDR |
| CRKP250 | no | no | R | R | R | R | R | R | R | R | R | R | R | R | R | R | R | R | R | S | R | no | no | yes | no | no | XDR |
| CRKP251 | no | no | R | R | R | R | R | R | R | R | R | R | R | R | S | R | R | R | R | S | R | no | no | yes | yes | no | XDR |
| CRKP252 | yes | yes | R | R | R | R | R | R | R | R | R | R | R | R | R | R | R | S | I | S | R | no | yes | yes | yes | no | XDR |
| CRKP253 | no | no | R | R | R | R | R | R | R | R | R | R | R | R | R | I | R | R | R | S | R | no | no | yes | no | no | XDR |
| CRKP255 | no | no | R | R | R | R | R | R | R | R | R | R | R | R | R | R | R | I | R | S | S | no | no | yes | no | no | XDR |
| CRKP257 | yes | yes | R | R | R | R | R | R | R | R | R | R | R | S | S | S | R | I | I | S | R | no | no | yes | yes | no | XDR |
| CRKP258 | no | no | R | R | S | R | R | S | R | R | R | S | S | S | S | S | R | S | S | S | R | no | no | yes | no | no | MDR |
| CRKP259 | no | no | R | R | R | R | R | R | R | R | R | R | R | R | R | I | R | R | R | S | R | no | no | yes | no | no | XDR |
| CRKP260 | yes | no | R | R | R | R | R | R | R | R | R | R | R | R | S | R | R | S | R | S | R | no | no | yes | no | no | XDR |
| CRKP261 | yes | no | R | R | R | R | R | R | R | R | R | R | R | S | S | R | R | S | R | S | R | no | no | yes | no | no | XDR |
| CRKP262 | no | no | R | R | R | R | R | R | R | R | R | R | R | R | S | S | R | S | R | S | S | no | no | yes | no | no | MDR |
| CRKP263 | yes | no | R | R | R | R | R | R | R | R | R | I | I | S | S | R | R | S | I | S | R | no | no | yes | no | no | XDR |
| CRKP264 | no | no | R | R | R | R | R | R | R | R | R | R | R | R | R | R | R | S | R | S | R | no | no | yes | no | no | XDR |
| CRKP265 | yes | no | R | R | R | R | R | R | R | R | R | R | R | S | S | S | R | R | R | S | S | no | no | yes | no | no | MDR |
| CRKP266 | no | yes | R | R | R | R | R | R | R | R | R | R | R | R | I | R | R | S | I | S | R | no | no | yes | no | no | XDR |
| CRKP267 | no | no | R | R | R | R | R | R | R | R | R | R | R | R | S | R | R | R | R | S | R | no | no | yes | yes | no | XDR |
| CRKP268 | no | no | R | R | R | R | R | R | R | R | R | R | I | R | S | R | R | S | R | S | R | no | no | yes | no | no | XDR |
| CRKP269 | yes | no | R | R | R | R | R | R | R | R | R | R | I | R | S | S | R | S | R | S | R | no | no | yes | no | no | MDR |
| CRKP270 | no | no | R | R | R | R | R | R | R | R | R | R | R | R | S | S | R | R | R | S | S | no | no | yes | no | no | MDR |
| CRKP271 | no | no | R | R | R | R | R | R | R | R | R | R | I | R | S | S | S | S | R | S | R | no | no | yes | no | no | MDR |

1. CLSI 2020 resistant breakpoints were used. Antimicrobial susceptible (S) and non-susceptible include intermediate (I) or resistant (R) rates * Categorized as MDR, XDR or PDR according to standard criteria. AMP; Ampicillin, AMC; Amoxicillin/Clavulanic acid, TZP; Piperacillin/tazobactam, CXM1; Cefuroxime, CXM2; Cefuroxime Axetil, FOX; Cefoxitin, CTX; Cefotaxime, CAZ; Ceftazidime, FEP; Cefepime, ETP; Ertapenem, IPM; Imipenem, MEM; Meropenem, AMK; Amikacin, GEN; Gentamicin, CIP; Ciprofloxacin, TGC; Tigecycline, NIT; Nitrofurantoin, CST; Colistin, SXT; Trimethoprim/Sulfamethoxazole. [↑](#footnote-ref-1)
